# Editorial: IPPS 2022 - plant phenotyping for a sustainable future

**DOI:** 10.3389/fpls.2024.1383766

**Published:** 2024-02-27

**Authors:** Elias Kaiser, Philipp Von Gillhaussen, Jennifer Clarke, Ulrich Schurr

**Affiliations:** ^1^ Horticulture and Product Physiology, Plant Sciences Group, Wageningen University and Research, Wageningen, Netherlands; ^2^ Forschungszentrum Jülich GmbH IBG-2: Plant Sciences, International Plant Phenotyping Network (IPPN), Juelich, Germany; ^3^ Quantative Life Sciences Initiative (Complex Biosystems), University of Nebraska-Lincoln, Lincoln, NE, United States; ^4^ Plant Sciences (IBG-2), Institute of Bio- and Geosciences, Julich Research Center, Helmholtz Association of German Research Centres (HZ), Juelich, Germany

**Keywords:** plant phenotyping, image analysis, model, phenomics, plant-environment interaction

Plants are a venue for addressing the challenges facing humanity. The need for a reliable supply of food, feed, materials, chemicals and energy as well as ways to manage agroecology and climate change are among the challenges that we can address through the sustainable use of plants and plant ecosystems. The research community needs to integrate plant systems approaches, from molecular to organismal to applications in the field and ecosystems, to increase productivity sustainably while using fewer land, water, and nutrient resources. In the past two decades, plant phenotyping research has developed a highly valuable portfolio of technologies, processes and infrastructures to address these questions (Pieruschka and Schurr, 2019). In the past, the creation of datasets was limited by low throughput sensing and image analysis (Tsaftaris et al., 2016). However, through the development of digital image analysis the previous phenotyping “bottleneck” has shifted towards a capacity problem, making it difficult to interpret vast datasets (especially in the face of plant x environment interactions), leading to an “interpretation bottleneck” (Smith et al., 2021). Innovative plant phenotyping approaches that reveal and target relevant traits are thus still needed to identify and quantify key traits and processes and to understand the dynamic interactions between genetics, molecular and biochemical processes, and the physiological responses to changes in the environment that lead to the development of a phenotype.

The IPPS 2022 conference in Wageningen (the Netherlands) brought together a diverse phenotyping community from academia and industry to discuss and realize potentials to harness the power of plant phenotyping. In this Research Topic (RT), we have collected contributions from attendees of IPPS 2022, as well as from other scientists working on plant phenotyping. The RT comprises ten experimental and three review papers. It is noteworthy that eight out of ten research papers are devoted to field crops (including the major crops wheat, maize, potato, sugarcane, and cotton), highlighting the community’s increasing focus on the application of plant phenotyping for crop improvement and the understanding of physiological patterns in large populations of crops for food, feed, and energy security. Plant phenotyping is a highly interdisciplinary field, as it requires constant development and critical evaluation of methods in both data acquisition and analysis. The papers of this RT can be categorized broadly into those focused on data collection (7 papers), those focused on data analysis and/or modeling (5 papers), and one review paper on policy and governance that broadly deals with both aspects (Gerullis et al.).

Regarding data collection through rapid phenotyping, several authors applied existing methods to new problems, thereby expanding the tested range of these methods. Ma et al. successfully applied near-infrared spectroscopy to a diversity panel of sugarcane to detect differences in stalk crushing strength, a trait closely related to mechanical stability of sugarcane. Using this method, breeders may be able to breed for more lodging-resistant sugarcane. In a noteworthy example of phenotyping of growth and photosynthesis during the growing season in the field, Knopf et al. assessed the genotypic diversity of ten wheat cultivars under ambient and elevated (CO_2_). Among other sensors, the light-induced fluorescence transient (LIFT) sensor was used, enabling the researchers to detect earlier onset of senescence under elevated (CO_2_). Shi et al. provide an example of combined phenotyping of root and shoot growth in maize, an approach that is currently unusual and deserves more attention given the intimate connection of root and shoot functioning, as well as the importance of above- and belowground biomass allocation. Njane et al. assessed the effects of UAV height on imaging of potato, for traits including crop height and volume. They determined that a flying height of 15 m was preferable to that of 30 m, as it provided for better resolution. Dong et al. visually inspected seeds of several accessions of the leguminous plant *Sophora moorcroftiana*, identifying genetic variation in traits that in other species have been shown to correlate with fitness in the field, such as seed weight, providing implications for crop improvement in legumes, which contribute largely to global food security. In their review paper on Sainfoin (*Onobrychis* spp. Fabaceae), Karabulut et al. provide an overview of all traits (82 in total) which have so far been measured on this perennial forage legume, which is mostly used as livestock feed but could feed humans as well.

Although they are undoubtedly useful, large high-throughput phenotyping (HTP) facilities are subject to several pitfalls, as illustrated in the review by Poorter et al. For example, projected leaf area, which is often used to estimate biomass, can be underestimated by ~20% due to diurnal leaf movement. Also, Poorter et al. highlight the fact that the high degree of automation that HTP systems require results in reduced experimental flexibility (in terms of possible measurements and treatments) and a demand for expert knowledge (to run and fix such systems). Proxies generated by such systems often require calibration curves that are specific to a given crop. Given the inflexibility in the set of traits measured by many HTP systems, researchers using such systems may fall prey to the “if the only tool you have is a hammer, everything looks like a nail” problem. The importance of systemic approaches to regulation and governance in plant breeding is highlighted by Gerullis et al. The authors propose a new governance heuristic – a rule of thumb for decision makers – for evaluating plant breeding research that includes social systems feedback, along with genetics, environment and management.

Several publications report progress on the use of data analysis and modelling for trait estimation. One highlight is presented by Cantürk et al. who used 3D point clouds based on RGB and laser data acquired by UAVs to detect key morphological features of vine plants in the field, including plant height, plant volume and canopy width. Key to determining these features was correct identification of trunk location, which allowed for the identification of single plants. Carlier et al. tested several model types on RGB and multispectral data of wheat, identifying convolutional neural network (CNN) models to be superior to partial least squares regression (PLSr) models for trait extraction. Similarly, Renó et al. used two AI models – random forest and multilayer perceptron processing – to detect drought in cotton using thermography, thereby increasing the throughput of thermal image analysis.

The last two papers of this RT deal with the connection between phenomics data and genetics, a topic that is highly relevant for plant breeding. In a population of potato grown throughout several seasons and across various levels of heat stress, Martins et al. showed that including a family effect significantly improved the genetic selection of potato clones for subsequent breeding. Finally, Li et al. describe an interesting example of using phenomic rather than genomic selection to estimate genetic diversity in Scots pine. They performed phenomic selection using hyperspectral reflectance data acquired by UAVs, which in many cases is much easier and cheaper to obtain than molecular markers, especially in long-living woody plants. Phenomic selection may hold great promise in the future of plant breeding.

We believe that this RT is a nice representative sample of the state of the art of plant phenotyping. We hope that readers will thoroughly enjoy these articles and derive valuable knowledge from them.

## Author contributions

EK: Writing – original draft, Writing – review & editing. PG: Writing – original draft, Writing – review & editing. JC: Writing – original draft, Writing – review & editing. US: Writing – original draft, Writing – review & editing.
